# Vitamin D in Autoimmunity: Molecular Mechanisms and Therapeutic Potential

**DOI:** 10.3389/fimmu.2016.00697

**Published:** 2017-01-20

**Authors:** Wendy Dankers, Edgar M. Colin, Jan Piet van Hamburg, Erik Lubberts

**Affiliations:** ^1^Department of Rheumatology, Erasmus MC, University Medical Center, Rotterdam, Netherlands; ^2^Department of Immunology, Erasmus MC, University Medical Center, Rotterdam, Netherlands; ^3^Department of Rheumatology, ZGT, Almelo, Netherlands

**Keywords:** vitamin D, autoimmune disease, supplementation, T cells, B cells, dendritic cells, macrophages

## Abstract

Over the last three decades, it has become clear that the role of vitamin D goes beyond the regulation of calcium homeostasis and bone health. An important extraskeletal effect of vitamin D is the modulation of the immune system. In the context of autoimmune diseases, this is illustrated by correlations of vitamin D status and genetic polymorphisms in the vitamin D receptor with the incidence and severity of the disease. These correlations warrant investigation into the potential use of vitamin D in the treatment of patients with autoimmune diseases. In recent years, several clinical trials have been performed to investigate the therapeutic value of vitamin D in multiple sclerosis, rheumatoid arthritis, Crohn’s disease, type I diabetes, and systemic lupus erythematosus. Additionally, a second angle of investigation has focused on unraveling the molecular pathways used by vitamin D in order to find new potential therapeutic targets. This review will not only provide an overview of the clinical trials that have been performed but also discuss the current knowledge about the molecular mechanisms underlying the immunomodulatory effects of vitamin D and how these advances can be used in the treatment of autoimmune diseases.

## Introduction

Autoimmune diseases, including rheumatoid arthritis (RA), multiple sclerosis (MS), and Crohn’s disease (CD), result from an aberrant activation of the immune system, whereby the immune response is directed against harmless self-antigens. This results in inflammation, tissue damage, and loss of function of the affected organs or joints. With the increasing prevalence of autoimmunity in the Western countries (1), the societal burden of these diseases also increases. Although the treatment of autoimmune diseases has improved due to the development of so-called biologics, like tumor necrosis factor alpha (TNFα) inhibitors, a large proportion of patients are still not adequately responding to these treatments (2). Therefore, it is still important to improve current therapies or to uncover new treatment options.

In this context, the immunomodulatory effects of vitamin D provide opportunities to enhance the treatment of autoimmune diseases. First, given the high prevalence of vitamin D deficiency in patients suffering from autoimmunity, vitamin D supplementation might decrease disease severity or augment the therapeutic effect of current medication. Second, knowing the molecular mechanisms underlying the immunomodulatory effects could lead to the discovery of new potential therapeutic targets. Therefore, this review will explore the advances that have been made in both clinical trials and molecular studies. In addition, it will give an overview of the challenges that still remain before the immunomodulatory effects of vitamin D can be utilized in clinical practice.

## Vitamin D Metabolism, Signaling, and Function

**Figure 1:**
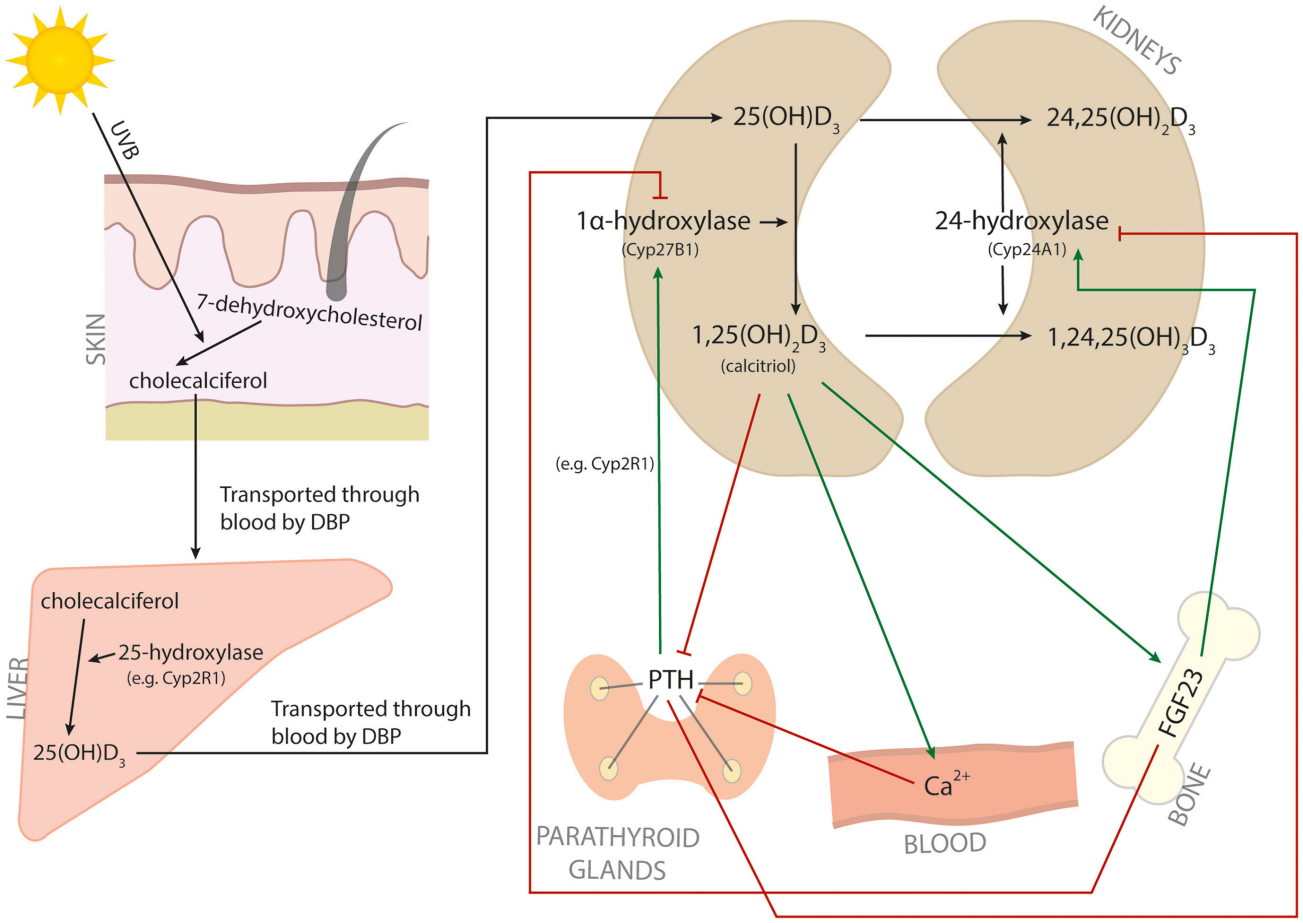
**Vitamin D metabolism**. The metabolic pathway of vitamin D. Red arrows indicate inhibition, and green arrows indicate induction.

Vitamin D, or cholecalciferol, is a secosteroid hormone that can be obtained from dietary sources, but that is predominantly synthesized in the skin from 7-dehydroxycholesterol in response to UV light (Figure 1). Cholecalciferol is bound by vitamin D-binding protein (DBP) and transported to the liver. In the liver, various cytochrome p450 (Cyp) vitamin D hydroxylases convert cholecalciferol into 25(OH)D_3_. Cyp2R1 is considered to be the primary 25-hydroxylase responsible for this process. Subsequently, DBP transports 25(OH)D_3_ to the kidneys, where the 1α-hydroxylase Cyp27B1 converts 25(OH)D_3_ into 1,25(OH)_2_D_3_. 1,25(OH)_2_D_3_, also called calcitriol, is the active vitamin D metabolite. To control calcitriol concentrations, the 24-hydroxylase Cyp24A1 hydroxylates 25(OH)D_3_ or 1,25(OH)_2_D_3_ at C-24, yielding the less active metabolites 24,25(OH)_2_D_3_ and 1,24,25(OH)_3_D_3_, respectively (3). The level of 1,25(OH)_2_D_3_ is therefore mainly determined by the balance between Cyp27B1 and Cyp24A1. Two proteins that are important for regulating this balance are fibroblast growth factor 23 (FGF23) and parathyroid hormone (PTH). FGF23 shifts the balance toward Cyp24A1 and therefore inactivation of vitamin D signaling, and is induced by high concentrations of 1,25(OH)_2_D_3_ and low serum phosphate. On the other hand, PTH favors the balance toward Cyp27B1 and activation of vitamin D signaling. PTH is inhibited by high concentrations of 1,25(OH)_2_D_3_ and induced by low serum calcium (3) (Figure 1).

1,25(OH)_2_D_3_ initiates its signaling cascade by binding to the vitamin D receptor (VDR), which is a nuclear receptor that acts as a transcription factor. VDR binds to vitamin D responsive elements (VDREs) in the DNA, mostly to so-called DR3-type VDREs that are characterized by two hexameric core binding motifs separated by three nucleotides. In the absence of ligand, VDR is mostly bound to non-DR3-type VDREs and is associated with corepressor proteins. When 1,25(OH)_2_D_3_ binds to VDR, this induces a conformational change leading to the formation of two new protein interaction surfaces. One is for binding with heterodimeric partners to facilitate specific DNA binding, such as retinoid X receptor (RXR), and the other is for recruitment of co-regulatory complexes that will exert the genomic effects of VDR (4). Furthermore, there is a shift in binding to primarily DR3-type VDREs (5). Interestingly, although RXR has multiple binding partners, specifically with VDR it will bind to the DR3-type elements. This indicates that the heterodimerization of VDR and RXR is important for functioning of the VDR (6). However, research in colorectal cancer cells has shown that 25% of the VDR binding sites are not enriched for RXR (7). No direct data on colocalization of VDR and RXR in immune cells have been reported, although Handel et al. found a significant overlap between VDR in CD4^+^ T cells and RXR in a promyelocytic leukemia cell line (8). Therefore, it is currently unknown whether the rate of VDR/RXR colocalization differs between cell types. Also, the functional consequence of VDR binding with or without RXR remains to be understood.

The best known function of 1,25(OH)_2_D_3_ is the maintenance of calcium homeostasis by facilitating the absorption of calcium in the intestine. However, in the presence of low 1,25(OH)_2_D_3_ levels, calcium will be mobilized from the bone rather than the intestine. If these conditions are prolonged, this may lead to osteomalacia and rickets, both well-known clinical signs of vitamin D deficiency. An overview of the current knowledge on the role of vitamin D signaling in calcium homeostasis was recently given by Carmeliet et al. and will not be discussed here (9). The first hint that vitamin D might also be important for extraskeletal health came from mycobacterial infections such as tuberculosis, in which vitamin D was used as a treatment before antibiotics were discovered (10). The discovery that the VDR is expressed in almost all human cells has further increased the attention for the extraskeletal effects of vitamin D. As a result, vitamin D deficiency has now been linked to not only bone health but also, for example, cancer, cardiovascular diseases, and autoimmune diseases (9).

## Vitamin D and Autoimmune Diseases

Since the discovery of the VDR on blood lymphocytes (11, 12), the effects of vitamin D on the immune system and immune-related diseases became the subject of a large number of studies. In this context, it was discovered that supplementation with 1,25(OH)_2_D_3_ could prevent both the initiation and progression of experimental autoimmune encephalomyelitis (EAE) and collagen-induced arthritis (CIA), experimental models of MS and RA, respectively (13–15). In addition, VDR deficiency aggravated arthritis severity in human TNFα transgenic mice (16). Similarly, vitamin D deficiency increased enterocolitis severity in IL-10 knock-out (KO) mice, which are used as a model system for inflammatory bowel diseases (IBDs). Treatment with 1,25(OH)_2_D_3_ decreased disease symptoms in both the IL-10 KO mice and in the dextran sulfate sodium (DSS)-induced colitis model (17, 18). Finally, treatment with 1,25(OH)_2_D_3_ reduced the incidence of diabetes in non-obese diabetic (NOD) mice (19, 20) and the severity of systemic lupus erythematosus (SLE) in MRL/1 mice (21).

These studies in experimental autoimmune models underscore the need to examine whether there is a protective role for vitamin D in human autoimmune diseases. In the last few decades, numerous studies have investigated the link between vitamin D and the incidence and severity of autoimmune diseases. One of the first indications was the correlation between increasing MS prevalence and increasing latitude, and consequently with decreasing sunlight exposure. Exceptions to this gradient can at least partially be explained by genetic variants (like the HLA-DRB1 allele) or lifestyle differences, such as high fish consumption (22). The relation between latitude and disease prevalence was also found for other autoimmune diseases such as type I diabetes mellitus (T1D) and IBD (23, 24). Further strengthening the link between sun exposure and autoimmunity is the finding that the risk of developing MS is correlated with the month of birth, with for the northern hemisphere a higher risk in April and a lower risk in October and November (25, 26). Importantly, this correlation can only be found in areas where the UV exposure changes during the year (25).

Next to UV exposure, vitamin D can also be obtained from dietary sources and supplements. A meta-analysis by Song et al. found that the incidence of RA is inversely correlated with vitamin D intake, both when considering dietary intake and supplements or supplements alone (27). In addition, vitamin D supplementation in early childhood might reduce the risk of developing T1D up to 30% depending on the supplementation frequency (28, 29). Also the effect of maternal vitamin D intake on the risk of T1D in the offspring has been investigated, but due to the limited amount of studies there is currently not sufficient evidence to prove a correlation (29).

Investigating the correlation between vitamin D intake and prevalence of autoimmunity is challenging because the measurements of dietary intake and UV exposure are often based on estimations. Therefore, it might be more useful to analyze the correlation between the serum 25(OH)D_3_ level and autoimmunity. Indeed, in many autoimmune diseases, patients have a lower serum 25(OH)D_3_ than healthy controls (30–36). In addition, patients with a lower 25(OH)D_3_ level are implicated to have higher disease activity (32, 35, 37). Although it is not clear whether the lower 25(OH)D_3_ level also increases the risk of autoimmunity, the study by Hiraki et al. suggested that there is a strong correlation between the risk of developing RA and the 25(OH)D_3_ level between 3 months and 4 years before diagnosis (38). It should be noted that all these studies merely demonstrate correlations, so it is still under debate whether the low 25(OH)D_3_ level is the cause or the result of the autoimmune disease.

Another line of evidence that indicates a role for vitamin D in human autoimmunity is the correlation with polymorphisms in the VDR. There are four well-known VDR polymorphisms that have been extensively studied for their potential role in autoimmunity: *Apa*I, *Bsm*I, *Taq*I, and *Fok*I. All of these polymorphisms have been associated with the risk of developing an autoimmune disease, although it differs between diseases and polymorphisms whether it is protective or a risk factor. Also, ethnicity plays a role in the correlation between the polymorphisms and autoimmune diseases (39–47).

In summary, autoimmune diseases are correlated with 25(OH)D_3_ serum levels, vitamin D intake, UV exposure, and VDR polymorphisms. Furthermore, 1,25(OH)_2_D_3_ suppresses disease in experimental autoimmune models. Although these data do not prove a causal relationship between vitamin D and autoimmune diseases, they warrant further investigation into whether at-risk individuals and patients could benefit from vitamin D supplementation.

## Vitamin D as a Therapeutic Agent in Human Autoimmune Diseases

Despite the beneficial effects of 1,25(OH)_2_D_3_ supplementation in experimental autoimmune models, the application of vitamin D derivatives in clinical practice is currently limited to topical use for the treatment of psoriasis (48). The systemic use of vitamin D in the treatment of other autoimmune diseases is still under investigation. Table 1 gives an overview of the placebo-controlled clinical trials investigating the effect of vitamin D supplementation in autoimmune diseases other than psoriasis. Here, we discuss these trials and what this means for the therapeutic potential of vitamin D in each of these autoimmune diseases.

**Table 1:** **Overview of randomized controlled trials with vitamin D supplementation in autoimmune diseases**.

Trial	Disease	Trial design	Inclusion criteria	Groups	Supplementation dosage	Supplemental calcium	Other medication	Baseline 25(OH)D_3_ in treated group (nmol/L)	Endpoint 25(OH)D_3_ in treated group (nmol/L)	Main clinical findings
Burton et al. (49)	Multiple sclerosis (MS)	Open-label RCT, 52 weeks	MS without a relapse within 60 days EDSS 0–6.5 Serum 25(OH)D_3_ < 150 nmol/L	*N* = 25 cholecalciferol, *N* = 24 placebo	Dose escalation: up to 280,000 IU/week in 23 weeks, stay 6 weeks, then reduce to 0 in 20 weeks, then 3 weeks without	1,200 mg daily	Continuation of MS medication, placebo-treated patients could take up to 4,000 IU cholecalciferol and supplemental calcium if desired. In case of relapse, patients received steroids as judged by the treating physician	80	Up to 400 nmol/L after the peak of dosage, 200 nmol/L at the end of the trial	Lower proportion of patients with an increase in EDSS at the end of the trial Trend toward reduced relapse rate

Mosayebi et al. (50)	MS	Double-blind RCT, 6 months (October–March)	MS with a relapse in the last year More than three lesions on MRI. EDSS 0–3.5	*N* = 28 cholecalciferol, *N* = 34 placebo	300,000 IU monthly (intramuscular)	No	IFNB-1a	25	150	No effect on EDSS No effect on Gd-enhancing lesions

Soilu-Hänninen et al. (51)	MS	Double-blind RCT, 12 months	RRMS with at least 1 month IFNB-1b treatment Serum 25(OH)D_3_ < 85 nmol/L	*N* = 34 cholecalciferol, *N* = 32 placebo	20,000 IU weekly	No	IFNB-1b	54	110	Reduced number of Gd-enhancing lesions, but no effect on other MRI parameters Trend toward reduced EDSS

Kampman et al. (52)	MS	Double-blind RCT, 96 weeks	MS with an EDSS < 4.5	*N* = 35 cholecalciferol, *N* = 33 placebo	20,000 IU weekly	500 mg daily	46% of patients in both groups were treated with IFNβ, 3% with glatiramer acetate and 3% in the placebo group with natalizumab	55	123	No effects on EDSS, relapse rate, function, or fatigue

Derakhshandi et al. (53)	MS	Double-blind pilot RCT, 12 months	Optic neuritis patients without MS	*N* = 13 cholecalciferol, *N* = 11 placebo	50,000 IU weekly, when reaching serum 25(OH)D_3_ of 250 nmol/L switch to a maintenance dose	No	3 × 1 g methylprednisolone/day i.v., then oral prednisolone	38	Unknown	Decreased incidence rate ratio of demyelinating plaques Reduced risk of progression to MS

Salesi and Farajzadegan (54)	Rheumatoid arthritis (RA)	Double-blind RCT, 12 weeks	RA with DAS28 > 3.2 At least 24 weeks MTX treatment	*N* = 50 25(OH)D_3_, *N* = 48 placebo	50,000 IU weekly	No	MTX Prednisone, HCQ, and CQ were allowed	107	125	Modest, non-significant, improvement in tender joint count, swollen joint count, ESR, and VAS

Dehghan et al. (55)	RA	Double-blind RCT, 6 months	RA in remission for at least 2 months Serum 25(OH)D_3_ < 75 nmol/L	*N* = 40 cholecalciferol, *N* = 40 placebo	50,000 IU weekly	No	Prednisone, MTX, and HCQ allowed	<75	Unknown	Non-significant decrease in relapse rate

Hansen et al. (56)	RA	Double-blind RCT 12 months	RA Serum 25(OH)D_3_ between 15.25 and 62.25 nmol/L	*N* = 11 cholecalciferol, *N* = 11 placebo	4 weeks: 50,000 IU 3× weekly 11 months: 50,000 IU 2× monthly When serum was below 62.5 nmol/L: 50,000 IU weekly for 8 weeks	500 mg 3× daily	SPF65	63	75 (after 2 months)	No effects on DAS28, HAQ, or physician global assessment of RA Non-significant increase in pain Increased patient assessment of global health and patient global assessment of RA

Jørgensen et al. (57)	Crohn’s disease (CD)	Double-blind RCT, 1 year	CD in remission (CDAI < 150) for at least 4 weeks	*N* = 46 cholecalciferol, *N* = 48 placebo	1,200 IU daily	1,200 mg daily	Azathioprine (39–44% of participants)	70	95	Trend toward reduced relapse (hazard ratio of 0.44)

Wingate et al. (58)	CD	Double-blind RCT, 6 months	Children with quiescent CD	*N* = 352,000 IU cholecalciferol, *N* = 34,400 IU cholecalciferol	400 or 2,000 IU daily depending on randomization	No	Multivitamins (without vitamin D) Normal inflammatory bowel diseases (IBD) medication (36% 5-ASA, 57% immunomodulator, 30% biologics)	63	70 (400 IU) or 86 (2,000 IU)	No difference between the groups in CDAI, ESR, or CRP

Raftery et al. (59)	CD	Double-blind RCT, 3 months	Adults with CD in remision (CDAI < 150) and stable therapy for 3 months	*N* = 13 cholecalciferol, *N* = 14 placebo	2,000 IU daily	Only when already on it for bone health	Normal IBD medication (51% 5-ASA, 67% immunomodulator, 7% anti-TNFα)	70	90	Intestinal permeability was stable in the treated group, but increased in the placebo group Reduced CRP, increased QoL and trend toward decreased CDAI in patients with serum 25(OH)D_3_ > 75 nmol/L

Li et al. (60)	T1D	Prospective RCT, 12 months	LADA patients with diagnosis <5 years	*N* = 17 alfacalcidol, *N* = 18 unsupplemented	0.25 µg twice daily	No	Insulin therapy in both groups	63	Unknown	Stable FCP while decline in control group, same trend for PCP. Especially pronounced when disease duration <1 year

Bizzarri et al. (61)	T1D	Double-blind RCT, 24 months	Recent-onset T1D	*N* = 15 calcitriol, *N* = 12 placebo	0.25 µg daily	No	Insulin therapy in both groups	<50	+3.9%	After 12 months, the decline in FCP is slower in treated group, but not anymore after 24 months

Walter et al. (62)	T1D	Double-blind RCT, 18 months	Adults with recent-onset T1D	*N* = 20 calcitriol, *N* = 18 placebo	0.25 µg daily	No	Insulin therapy in both groups	25 pg/mL [1,25(OH)D_3_]	30 pg/mL [1,25(OH)D_3_]	No changes in C-peptide or insulin dose

Gabbay et al. (63)	T1D	Double-blind RCT, 18 months	Patients with recent-onset T1D (age >7 years)	*N* = 17 cholecalciferol, *N* = 19 placebo	2,000 IU daily	No	Insulin therapy in both groups	65	150	Decreased progression to undetectable C-peptide Enhanced stimulated C-peptide after 12 months Decreased decay of stimulated C-peptide after 18 months

Ataie-Jafari et al. (64)	T1D	Single-blind RCT, 6 months	Patients with recent-onset T1D	*N* = 29 alfacalcidol, *N* = 25 placebo	0.25 µg once daily, or twice if blood calcium levels allowed it	No	Insulin therapy in both groups	32.5	Unknown	Better preservation of C-peptide and lower insulin dose. Stronger effect in males than in females

Abou-Raya et al. (65)	Systemic lupus erythematosus (SLE)	Double-blind RCT, 12 months	SLE with SLEDAI >1 Serum 25(OH)D_3_ < 75 nmol/L	*N* = 158 cholecalciferol, *N* = 89 placebo	2,000 IU daily	Yes, unknown dose	6% corticosteroids, 80% antimalarials, 26% AZA, 27% ACE inhibitors/ARB	50	98	Decrease in SLEDAI and ESR

Lima et al. (66)	SLE	Double-blind RCT, 24 weeks	Juvenile onset SLE SLEDAI < 12	*N* = 20 cholecalciferol, *N* = 20 placebo	50,000 IU weekly	No	Unknown, but stable during trial	50	78	Decrease in SLEDAI, trend to decrease in ECLAM and decrease of fatigue related to social life

Aranow et al. (67)	SLE	Double-blind RCT, 12 weeks	Adult SLE with IFNα signature Stable inactive disease Anti-dsDNA positive Serum 25(OH)D_3_ < 50 nmol/L	*N* = 184,000 IU cholecalciferol, *N* = 172,000 IU cholecalciferol, *N* = 19 placebo	2,000 IU or 4,000 IU daily	No	Unknown	28	75	No difference in IFN signature (based on three genes) or disease activity

*ASA, 5-aminosalicylzuur (sulfasalazine); CDAI, Crohn’s disease activity index; CQ, chloroquine; CRP, C-reactive protein; ECLAM, European consensus lupus activity measurement; EDSS, Expanded Disability Status Scale; ESR, erythrocyte sedimentation rate; FCP, fasting c-peptide; Gd, gadolinium; HAQ, health assessment questionnaire; HCQ, hydroxychloroquine; IU, International Units; LADA, latent autoimmune diabetes in adults; MTX, methotrexate; PCP, C-peptide after 75 g glucose; QoL, quality of life; RCT, randomized controlled trial; RRMS, relapsing-remitting multiple sclerosis; SLEDAI, systemic lupus erythematosus disease activity index; DAS28, disease activity score for 28 joints; VAS, visual analog scale*.

### Multiple Sclerosis

In the field of MS, several trials have been performed in which cholecalciferol was given to the patients, but the results are contradictory. Beneficial effects of cholecalciferol supplementation that have been reported include decrease in Expanded Disability Status Scale (EDSS), decrease in MRI lesions, increased functionality, and reduced relapse rates (49, 51). Importantly, cholecalciferol has an added effect when used as a supplement to interferon β (IFNβ) treatment (51). On the other hand, two other trials reported no difference in any of these parameters (50, 52). Vitamin D supplementation might also be important in the pre-MS stage, since cholecalciferol supplementation decreased the conversion rate of optic neuritis to chronic MS (53).

Due to the small sample size (no more than 35 patients/group) of these trials, it is difficult to draw conclusions from these data. Although the effect of cholecalciferol on conversion to chronic effect appears promising, this was only one study with 13 treated patients and 11 placebo controls. Therefore, more research is necessary to determine whether therapy with cholecalciferol is beneficial for MS patients.

### Rheumatoid Arthritis

Despite the beneficial effect of 1,25(OH)_2_D_3_ supplementation on experimental arthritis (15), there are to date only three randomized trials investigating the effect of supplementation on disease activity in RA. Although the studies performed by Salesi and Farajzadegan and Dehghan et al. suggested a beneficial effect on disease activity and relapse rate, respectively, neither results reach statistical significance (54, 55). However, Dehghan et al. pointed out that for every 10 patients treated with cholecalciferol, relapse would be prevented in one patient. Considering the costs and safety profile of cholecalciferol supplementation, this might be worth following up. Ergocalciferol, the less potent fungal equivalent of human cholecalciferol, had no effect on disease activity and was associated with worse patient-related health assessments (56). Similar to studies in MS, the major limitation in the three RA studies is the group size, which limits the power of the analyses. Therefore no definitive conclusion can be drawn yet whether vitamin D can be used as a therapeutic agent in RA.

### Crohn’s Disease

Crohn’s disease is a subtype of the IBDs and is investigated intensively for the effect of vitamin D supplementation. However, the difficulty with this disease is that the intestinal inflammation may lead to decreased absorption of the supplemented vitamin D. Nevertheless, for adult patients, cholecalciferol supplementation might reduce the risk of relapses, although the difference does not reach statistical significance (*p* = 0.06) (57). Correspondingly, cholecalciferol prevented further increase of intestinal permeability, which may be an early marker of relapse (59). This is even more pronounced when the patients are stratified based on their serum 25(OH)D_3_ level. Additionally, patients with a serum level above 75 nmol/L have significantly lower serum levels of C-reactive protein (CRP, a marker of inflammation) and a non-significant decrease in disease activity as measured with Crohn’s Disease Activity Index (59). These studies used 1,200–2,000 IU cholecalciferol daily in adults, but in children there is no difference in disease activity between supplementing 400 and 2,000 IU daily despite a serum 25(OH)D_3_ level that is 25 nmol/L higher in the latter group (58).

When compared to RA and MS, the results for adult CD are more consistently showing a beneficial effect of cholecalciferol treatment. Since group sizes are again small, more research is required to confirm these data.

### Type I Diabetes Mellitus (T1D)

In contrast to the other autoimmune diseases where cholecalciferol supplementation is investigated, in T1D almost all trials use 1,25(OH)_2_D_3_ or an analog. Both forms appear to delay, but not prevent, the progression of β cell destruction in three studies (60, 63, 64). On the other hand, no effect of 1,25(OH)_2_D_3_ on T1D was observed in studies performed by Bizzarri et al. (61) and Walter et al. (62). This lack of effect could be due to the low level of remaining β cell function at the start of the study, suggesting that the therapeutic window for vitamin D supplementation is in the earliest phases of the disease. The study by Li et al. found that the protective effect is only visible when the disease duration was less than 1 year, supporting this hypothesis (60). In T1D, the beneficial effects of 1,25(OH)_2_D_3_ may lie more in the prevention of disease onset (28, 29) than in the treatment of disease, since the destruction of β cells cannot be reversed.

### Systemic Lupus Erythematosus

Vitamin D supplementation in SLE might even be more relevant than in the other autoimmune diseases, since 80% of the patients is sensitive for sunlight and therefore protect themselves against UV exposure (68). Two studies supplementing either 2,000 IU daily or 50,000 IU weekly demonstrated decreasing disease activity score, auto-antibody levels, and fatigue (65, 66). Conversely, the type I interferon (IFN) signature was unchanged after 12 weeks of 2,000 or 4,000 IU cholecalciferol in another study (67). Since this study was performed in patients with inactive disease, had a short supplementation period, and the signature was based on the expression of only three genes, it remains to be determined whether cholecalciferol supplementation truly does not affect the complete IFN signature in patients with active disease.

Systemic lupus erythematosus is the only autoimmune disease is which a larger study was done, with 158 cholecalciferol-treated patients and 89 placebo controls (65). The promising results in this clinical trial await further confirmation before vitamin D can be used therapeutically in these patients.

## Immune Modulation by Vitamin D

In addition to exploring the potential of therapeutic vitamin D supplementation, there has been a great deal of research toward the working mechanisms of 1,25(OH)_2_D_3_ in cells of the immune system. Since autoimmune diseases are characterized by an overactive immune response, it seems logical that the beneficial effects of vitamin D on autoimmunity are due to effects on the immune system. Furthermore, virtually all immune cells express the VDR, making them susceptible to 1,25(OH)_2_D_3_-mediated modulation (11, 12, 69, 70). Various immune cells, including monocytes, dendritic cells, macrophages, B cells, and T cells, also have the capability to convert 25(OH)D_3_ into 1,25(OH)_2_D_3_ (71–78). This allows for local regulation of the concentration of 1,25(OH)_2_D_3_ at the site of inflammation and illustrates an important role for the cells of the immune system in the systemic effects of vitamin D.

**Figure 2:**
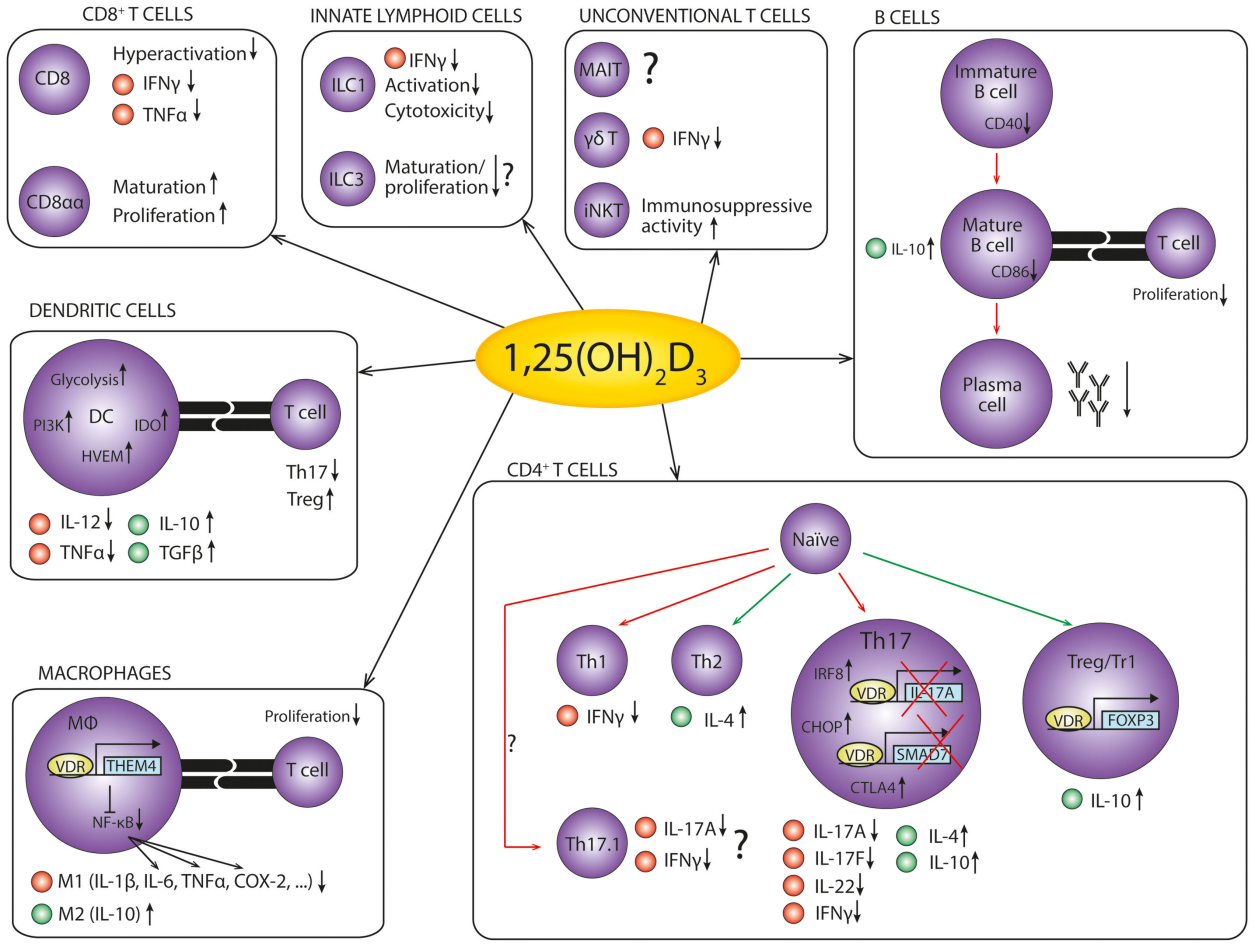
**The anti-inflammatory effects of 1,25(OH)_2_D_3_ on cells of the immune system**. An overview of the anti-inflammatory effects of 1,25(OH)_2_D_3_ on the cells of the immune system in autoimmunity. Red dots represent pro-inflammatory cytokines, while green dots represent anti-inflammatory cytokines. Red arrows indicate decreased differentiation, and green arrows indicate increased differentiation. References: CD8^+^ T cells (79–81); innate lymphoid cells (82–86); unconventional T cells (87–89); B cells (75, 90–96); dendritic cells (97–103); macrophages (104–108); CD4^+^ T cells (109–125).

Therefore, insight into how 1,25(OH)_2_D_3_ modulates the immune system could uncover new therapeutic targets in autoimmune diseases. Here, we discuss the effects of vitamin D on various cell types involved in the immune response, the current knowledge about the underlying mechanisms, and what this means for the therapeutic potential of vitamin D in autoimmunity (Figure 2).

### Dendritic Cells

Dendritic cells are antigen-presenting cells (APCs), which means that their main function is to take up foreign antigens and present them as peptides to T cells on the human leukocyte antigen (HLA) molecules. DCs are predominantly found in an immature state in peripheral tissues such as the skin, gut, and lungs, where they probe the surroundings for potential pathogens. Upon encountering a foreign antigen, they mature and migrate to the lymphoid tissues to stimulate antigen-specific T cells. Depending on the cytokines secreted by the DC, the T cell will differentiate into an effector cell with appropriate pro- or anti-inflammatory properties. Through these actions, APCs are crucial in initiating effective adaptive immune responses against pathogens, and also for maintaining self-tolerance and immune homeostasis.

The important role of DCs in autoimmune pathogenesis is illustrated in experimental autoimmune models, where deletion of specific DC subtypes ameliorates, or even prevents disease onset (126–129). In addition, APCs, including DCs and also macrophages and B cells, are associated with human autoimmunity through the correlation between specific HLA alleles and the risk of developing an autoimmune disease. For example, HLA-DRB1*15:01 is associated with an increased risk for MS (130), while HLA-DRB1*04:01 confers a greater susceptibility to RA (131).

Dendritic cells differentiated *in vitro* from monocytes or bone marrow cells in the presence of 1,25(OH)_2_D_3_ will remain in an immature-like tolerogenic state. This is characterized by decreased production of pro-inflammatory factors like IL-12 and TNFα and increased anti-inflammatory IL-10 production. These tolerogenic DCs (tDCs) are less capable of promoting proliferation and cytokine production of pro-inflammatory T cells, while they induce the differentiation of T regulatory (Treg) cells (97–99). Furthermore, they specifically induce apoptosis in autoreactive T cells, while not affecting proliferation of other T cells (132). Of note, 1,25(OH)_2_D_3_ can only induce this tolerogenic phenotype in DCs when it is added before their maturation. Once a maturation stimulus like lipopolysaccharide (LPS) is present or when the cells have already matured, the effects of 1,25(OH)_2_D_3_ on DCs are minimal (133). Aside from *in vitro* differentiated DCs, 1,25(OH)_2_D_3_ also induces a tolerogenic phenotype in dermal DCs, Langerhans cells, and plasmacytoid DCs, even though there are subtle differences between the effects on these subsets (100, 134, 135).

While the tolerizing effects of 1,25(OH)_2_D_3_ on DCs are well described, the underlying mechanisms are less clear. Recently, Ferreira et al. suggested that a metabolic switch toward glycolysis and activation of the PI3K-Akt-mTOR pathway are the first steps for the generation of tDCs by 1,25(OH)_2_D_3_ (101). Also the induction of indoleamine 2,3-dioxygenase (IDO) on DCs has been reported to be essential for the induction of a tDC phenotype and thereby for the beneficial effect of 1,25(OH)_2_D_3_ on EAE (102). Although all tDCs promote regulatory T cells (Tregs), the mechanism by which they do this depends on the type of DC. While tDC derived *in vitro* from bone marrow cells promote Tregs via induction of herpesvirus entry mediator (HVEM), tolerized Langerhans cells use TGFβ for this (100, 103). Dermal DCs induce the differentiation of T regulatory 1 (Tr1) cells, another type of Treg, via IL-10 (100). So in recent years, advances have been made to fully understand how 1,25(OH)_2_D_3_ modulates DCs, but the picture is not yet complete.

Despite the incomplete understanding of the molecular mechanism behind the effects of 1,25(OH)_2_D_3_ on DCs, tDCs generated with 1,25(OH)_2_D_3_ alone or in combination with dexamethasone are considered for therapy in autoimmune diseases (136). Their persistent tolerogenic state and the possibility to pulse them with tissue-specific antigens have made them valuable candidates to treat various diseases, including autoimmune diseases (99, 132, 137). This is illustrated in experimental disease models for T1D, MS, and RA, where administered antigen-specific tDCs migrate to inflammatory sites and reduce disease activity upon administration (102, 138–140). Importantly, DCs with an increased activation status from patients with autoimmune diseases can become equally tolerogenic in response to 1,25(OH)_2_D_3_ as healthy DCs (141–145). Because they can also be pulsed with auto-antigens and they can be generated under current Good Manufacturing Practice conditions, this opens up the way for the use of autologous tDCs in the treatment of human autoimmune diseases (141, 146). Currently, the use of tDCs generated with 1,25(OH)_2_D_3_ has not been clinically tested. However, tDCs generated using antisense oligonucleotides or Bay11-7082 were found to be safe upon administration in patients with T1D or RA, respectively (147, 148).

It remains to be determined whether these tDCs also have effects on disease activity and whether tDCs generated using 1,25(OH)_2_D_3_ could also be used in this context. Increased understanding on how 1,25(OH)_2_D_3_, with or without dexamethasone, modulates the DCs can provide insights in how to further optimize the tolerogenic potential of the DCs.

### Macrophages

Macrophages are known for their supreme phagocytic capacities, but they are also important APCs. In a normal immune response, an infection activates tissue-resident macrophages after which they produce inflammatory mediators and recruit other immune cells to eradicate the pathogen. Macrophages can roughly be divided into two categories: the M1 and M2 macrophages. M1 macrophages produce pro-inflammatory mediators like nitric oxide, TNFα, IL-23, IL-12, and IL-1β, whereby they kill pathogens and promote the polarization of T helper (Th) cells to T helper 1 (Th1) and Th17 cells to assist in the immune response. On the other hand, M2 macrophages produce the anti-inflammatory cytokine IL-10 and are important in wound repair and restoring tissue homeostasis (149).

The role of macrophages in the pathogenesis of autoimmune diseases is illustrated by an increase in macrophages at inflammatory sites (150–153). In addition, macrophages are hyperactivated and produce more pro-inflammatory cytokines, suggesting a dysregulated balance between M1 and M2 cells (104, 151, 154). As a result of their hyper-inflammatory state, they are essential for the development and activation of β-cell specific cytotoxic T cells, which leads to insulitis in NOD mice (155). Interestingly, the suppression of EAE by 1,25(OH)_2_D_3_ is preceded by a rapid reduction of macrophages in the CNS. This suggests that macrophages are another important target for vitamin D in the suppression of autoimmunity (156).

Notably, 1,25(OH)_2_D_3_ has dual roles in macrophage differentiation and activation. In the early stages of infection, 1,25(OH)_2_D_3_ stimulates differentiation of monocytes into macrophages (157). Furthermore, toll-like receptor triggering or IFNγ-induced activation activates Cyp27B1 and thereby potentiates the conversion of 25(OH)D_3_ into 1,25(OH)_2_D_3_ (158, 159). 1,25(OH)_2_D_3_ obtained via this pathway is then required for producing cathelicidin and for the antimicrobial activity of human monocytes and macrophages (160, 161). In addition, 1,25(OH)_2_D_3_ induces IL-1β, either directly or via upregulation of C/EBPβ or Erk1/2 (162, 163). So initially, 1,25(OH)_2_D_3_ is essential for effective pathogen clearance.

The hyperresponsiveness of VDR^−/−^ mice to LPS stimulation indicates that in the later stages of infection, 1,25(OH)_2_D_3_ plays a role in the contraction of the immune response (105). The anti-inflammatory effect of 1,25(OH)_2_D_3_ on macrophages is characterized by decreased production of pro-inflammatory factors such as IL-1β, IL-6, TNFα, RANKL, COX-2, and nitric oxide and increased anti-inflammatory IL-10 (104–108). These changes suggest that 1,25(OH)_2_D_3_ promotes the M2 phenotype while inhibiting the M1 phenotype, thereby restoring the balance between these subsets. Finally, 1,25(OH)_2_D_3_-treated macrophages have reduced T cell stimulatory capacity (108).

In recent years, some advances were made with unraveling the mechanism behind this anti-inflammatory effect of 1,25(OH)_2_D_3_ on macrophages. An important target of 1,25(OH)_2_D_3_ is thioesterase superfamily member 4 (THEM4), an inhibitor of the NFκB signaling pathway. THEM4 inhibits the direct binding of NFκB to the COX-2 locus and thereby prevents COX-2 transcription (106). Furthermore, THEM4 inhibits IL-6 and TNFα expression by preventing the signaling cascade in which NFκB induces miR-155 to suppress SOCS (105). Whether this THEM4-dependent pathway also inhibits the other pro-inflammatory mediators is not yet clear (104).

The balancing effect of 1,25(OH)_2_D_3_ between the pro- and anti-inflammatory status of macrophages is of particular interest in the treatment of autoimmune diseases. Currently, many inflammatory mediators secreted by M1 macrophages, like IL-1β, COX-2, IL-6, and especially TNFα, are already successful therapeutic targets in various autoimmune diseases. However, since current therapies result in systemic reduction of these mediators, patients may become prone to infections. Therefore, it is of interest to understand the mechanism by which 1,25(OH)_2_D_3_ balances between pro- and anti-inflammatory actions. This may provide insights in how to suppress the pro-inflammatory cytokines only in case of hyperactivation, without affecting the normal immune response.

### B Cells

B cells are mostly known for their crucial role in the immune response via the differentiation toward plasma cells and the production of antibodies. However, they also modulate the immune response via antigen presentation and cytokine secretion. In the context of autoimmunity, B cells play a crucial role by the production of autoreactive antibodies. These auto-antibodies, like anti-nuclear antibodies (ANAs) in SLE and anti-citrullinated peptide antibodies (ACPA) in RA, can be found in >95% and 70% of patients, respectively (164, 165).

Interestingly, the VDR binds to the promoter region of genes involved in the immune system in lymphoblastoid B cell lines, suggesting a role for B cells in the effect of vitamin D on autoimmune diseases (166). Here, we discuss what is known about the direct effects of 1,25(OH)_2_D_3_ on B cell differentiation and the three B cell functions of antibody production, cytokine secretion, and antigen presentation.

Before B cells become plasma cells that secrete high-affinity antibodies, they have to go through various stages of differentiation, class-switch recombination and somatic hypermutation (167). Various reports indicate that 1,25(OH)_2_D_3_ reduces the proliferation of B cells, induces their apoptosis and inhibits immunoglobulin class switching (90–92). This inhibition of differentiation may involve preventing nuclear translocation of NF-κB p65 and thereby inhibiting the signaling pathway downstream of CD40 costimulation (93). On the other hand, 1,25(OH)_2_D_3_ stimulates plasma cell development when added to terminally differentiating B cells. Furthermore, it induces the chemokine receptor CCR10 on these plasma cells, promoting their migration toward mucosal sites of inflammation (168). Therefore, it appears that the effect of 1,25(OH)_2_D_3_ depends on the activation and differentiation status of the B cells.

Independent of the effect of 1,25(OH)_2_D_3_ on B cell differentiation, there is ample evidence that it decreases the antibody production (90–92, 94, 95). Interestingly, the presence of ANA is correlated with a lower serum 25(OH)D_3_ level even in healthy people without SLE (169), while cholecalciferol supplementation decreases auto-antibody titers (65, 109).

Next to antibody production, B cells also secrete cytokines to influence the inflammatory milieu. Interestingly, VDR binds directly to the promoter region of IL-10 in B cells, thereby inducing the expression of IL-10 (75). However, in a cohort of healthy controls and relapsing-remitting MS patients, there was no correlation between IL-10 producing B cells and serum 25(OH)D_3_ levels (170).

There has been limited research toward the effect of 1,25(OH)_2_D_3_ on the APC function of B cells. However one study suggested that B cells primed with 1,25(OH)_2_D_3_ have decreased CD86 surface expression. Thereby, these B cells are less potent stimulators of naïve T cell proliferation and cytokine production (96).

Altogether, the effect of 1,25(OH)_2_D_3_ on B cells is still not completely clear. Currently, it is hypothesized that 1,25(OH)_2_D_3_ inhibits the pathogenic function of B cells in autoimmunity by preventing plasma cell differentiation and thereby auto-antibody production, by inducing IL-10 production and by inhibiting the antigen presentation capabilities. However, the limited amount of studies warrants further research to support this hypothesis and what role these effects play in the suppression of autoimmunity by 1,25(OH)_2_D_3_.

### T Cells

Historically, it was thought that DCs were the main target of vitamin D and that effects observed on T cells were mediated via DCs. However, it has now become clear that upon activation, various T cell populations express the VDR, including CD4^+^ Th cells, CD8^+^ cytotoxic T cells, and TCRγδ cells (12, 87, 171). This makes the T cell another direct immunological target for 1,25(OH)_2_D_3_. The effects of 1,25(OH)_2_D_3_ on T cells include modulation of cytokine secretion and differentiation, but VDR is also required for the activation of T cell by propagating TCR signaling (77). Since T cells are proposed to play an important role in the pathogenesis of autoimmunity, we will discuss the effects of 1,25(OH)_2_D_3_ on the various T cell populations.

#### CD4^+^ T Cells

CD4^+^ T cells are a heterogeneous group of cells, including Th1, Th2, Th17, and Treg cells. In the normal immune response, Th1 cells are important for fighting intracellular pathogens, Th2 cells for helminth infections and Th17 cells for extracellular pathogens and fungi. On the other hand, Tregs mediate immunological tolerance against self-antigens and harmless foreign antigens such as food and intestinal microbiota. Furthermore, they control the immune response via various mechanisms, including the secretion of anti-inflammatory mediators such as IL-10 and TGF-β (172). However, in autoimmune diseases, T cells mediate an immune response against the body itself, suggesting either hyperactivation of the pro-inflammatory T cells or insufficient control by Treg cells, or both.

The importance of the T cells as a target of 1,25(OH)_2_D_3_ in experimental autoimmune diseases is illustrated by Mayne et al., who showed that 1,25(OH)_2_D_3_ is not able to suppress EAE when the VDR is absent in T cells (173). For these studies, they used the CD4-Cre system, resulting in VDR deficiency in both CD4^+^ and CD8^+^ T cells. However, in this disease model, CD4^+^ T cells are likely the prime 1,25(OH)_2_D_3_ target cells, since other studies show that in this model CD8^+^ T cells are dispensable for the effects of 1,25(OH)_2_D_3_ (174). Further strengthening the hypothesis that the suppression of EAE by 1,25(OH)_2_D_3_ is driven by modulation of CD4^+^ T cells, is the finding that 1,25(OH)_2_D_3_ prevents CD4^+^ Th cell migration into the CNS (175). Finally, VDR binding is enriched near SNPs associated with autoimmune diseases in human CD4^+^ T cells, suggesting that these cells are also important in the effects of 1,25(OH)_2_D_3_ in human autoimmunity (8).

Because the effects of 1,25(OH)_2_D_3_ differ between the various CD4^+^ Th cell subsets (110), we will give an overview of the current knowledge on how these individual subsets are modulated by 1,25(OH)_2_D_3_ to suppress the autoimmune response.

##### Th1 and Th2 Cells

Classically, CD4^+^ T cells were subdivided into two classes: Th1 and Th2 cells. Th1 cells are characterized by the expression of IFNγ and T-bet, while Th2 cells produce IL-4, IL-5, and IL-13 and express the transcription factor GATA3. In the context of autoimmunity, it was long thought that Th1 cells mediate the disease pathogenesis, since mice lacking the transcription factor T-bet are protected against EAE (176). However, the discovery of Th17 cells, which will be discussed in the next section, and the finding that IFNγ is not required for induction of autoimmunity have led to a debate as to whether Th1 cells are important for autoimmune pathogenesis (177, 178). However, since adoptive transfer of myelin-specific IFNγ^+^ cells induces EAE (179), Th1 cells may still play a role in the disease pathogenesis.

Within Th1 cells, some studies suggest that 1,25(OH)_2_D_3_ inhibits IFNγ production when added at the first phases of differentiation (111, 180). On the other hand, another study found no effects on IFNγ (110). This contradiction could be explained by the addition of exogenous IL-2 in the first two studies. Since 1,25(OH)_2_D_3_ directly downregulates IL-2, exogenous IL-2 might be required for the inhibition of IFNγ by 1,25(OH)_2_D_3_ (181, 182). Although these studies indicate that 1,25(OH)_2_D_3_ modulates Th1 cells under certain circumstances, given their relatively small role in autoimmune pathogenesis and the low expression of VDR compared to other CD4^+^ T cell subsets, it is unlikely that they play an important role in the suppression of autoimmunity by 1,25(OH)_2_D_3_ (110, 112).

In contrast to Th1 cells, Th2 cells might be protective in Th17-driven autoimmune diseases even though they are pathogenic in the development of asthma and allergies. Studies in experimental arthritis demonstrate that T cell-specific overexpression of GATA3 is protective in autoimmunity due to suppression of Th17 responses (183). Interestingly, IL-4 is required for 1,25(OH)_2_D_3_ to inhibit EAE, suggesting an important role for this cytokine in the effect of 1,25(OH)_2_D_3_ (88). In the same model, 1,25(OH)_2_D_3_ induces GATA3 and its regulator STAT6. The functional relevance of this upregulation is demonstrated in STAT6-KO mice, where 1,25(OH)_2_D_3_ is unable to inhibit EAE development (184). Altogether these studies suggest a role for Th2 induction in the immune suppression by 1,25(OH)_2_D_3_.

However, the data on the effect of 1,25(OH)_2_D_3_ on Th2 cytokines like IL-4 seems contradictory. When naïve CD4^+^ T cells or the entire CD4^+^ T cell population are cultured without polarizing cytokines, 1,25(OH)_2_D_3_ induces IL-4 and GATA3 (113, 114). Also, in PBMC of treatment-naïve early RA patients, where IL-4 production is diminished, 1,25(OH)_2_D_3_ restores the IL-4 levels to the levels of healthy controls (115). However, when naïve CD4^+^ T cells, effector CD4^+^ T cells, or total CD4^+^ T cells are cultured in the presence of IL-4 to induce Th2 polarization, cellular IL-4 production is unaffected or even inhibited by 1,25(OH)_2_D_3_ (111, 180). Also when patients are supplemented with cholecalciferol, there is no increased IL-4 production by their T cells (109, 116, 117). Combining these data leads to the hypothesis that 1,25(OH)_2_D_3_ promotes Th2 differentiation and IL-4 production to assist in suppression of autoimmunity, but only when no sufficient IL-4 is present. The mechanism behind the precise regulation of IL-4 is of interest not only for treatment of autoimmunity but also of allergies and asthma where Th2 cytokines play an important pathogenic role.

##### Th17 Cells

In most autoimmune diseases, Th17 cells are considered to be important drivers of disease pathogenesis. Th17 cells are characterized by production of cytokines such as IL-17A, IL-17F, TNFα, and GM-CSF and the transcription factor RORC2 (RORγt in mice). They can also be distinguished based on the expression of the chemokine receptor CCR6, which directs migration toward the chemokine CCL20. Their differentiation can be driven by TGFβ, IL-6, and IL-1β, but they require IL-23 to become pathogenic Th17 cells (185). In 2003, two hallmark studies showed that IL-23, and not IL-12, is required for the induction of EAE and CIA (186, 187), suggesting an important role for the IL-23/IL-17 immune pathway in the pathogenesis of autoimmune diseases. Indeed, local IL-17A overexpression in mouse knee joints induces an arthritis-like phenotype with inflammation, bone erosions, and damaged cartilage (188). In EAE, the pathogenic cells appear to be the ex-Th17 cells, which now express IFNγ and T-bet, indicating the importance of Th17 plasticity in autoimmune diseases (189). In human autoimmunity, for example, in RA and SLE, levels of Th17 cells are elevated in the peripheral blood and synovial fluid of patients and correlate with disease activity (190–192). Furthermore, specifically the CCR6^+^ memory Th cells, which include Th17 cells, are potent activators of synovial fibroblasts (190). We have previously shown that this interaction leads to a pro-inflammatory feedback loop with increased production of IL-17A, IL-6, IL-8, and tissue-destructive enzymes. Via this mechanism, Th17 cells may contribute to local joint inflammation in RA (190). Combining the important role of Th17 cells in autoimmunity and the beneficial effect of 1,25(OH)_2_D_3_ on autoimmune diseases, it is hypothesized that 1,25(OH)_2_D_3_ suppresses autoimmunity at least partially via the inhibition of Th17 activity.

In support of this hypothesis, the effect of 1,25(OH)_2_D_3_ on an experimental model for antiretinal autoimmunity depends on inhibiting Th17 activity (193). Also *in vitro* 1,25(OH)_2_D_3_ decreases expression of pro-inflammatory cytokines like IL-17A, IL-17F, and IL-22 in CD4^+^ T cells, CD4^+^ memory cells, or CD4^+^CCR6^+^ memory cells (115, 118–120). Functionally, this decrease in Th17 activity diminishes activation of synovial fibroblasts, thereby inhibiting the pro-inflammatory loop between these cell types (120). Interestingly, 1,25(OH)_2_D_3_ also inhibits the secretion of IL-17A and other Th17 cytokines in the presence of Th17-polarizing cytokines (119, 121).

1,25(OH)_2_D_3_ not only inhibits the activity of Th17 cells but also Th17 differentiation. When naïve CD4^+^ T cells are differentiated toward the Th17 lineage *in vitro*, the presence of 1,25(OH)_2_D_3_ inhibits Th17-related cytokines and transcription factors such as IL-17A, IL-17F, RORC, and CCR6 (110, 112, 122). Functionally, MOG-specific Th17 cells differentiated in the presence of 1,25(OH)_2_D_3_ are less capable of inducing EAE upon adoptive transfer (119). Aside from the decreased pathogenicity of the cells, this effect may also be due to a decrease in CCR6, the chemokine receptor required for migration to the CNS (123).

Although the inhibitory effect on Th17 activity is well described, the mechanisms behind it are less clear. First of all, Joshi et al. showed that the regulation of IL-17A can be mediated via direct binding of the VDR to the IL-17A promoter. VDR–RXR complexes compete with NFAT for the binding sites in the promoter, after which they recruit RUNX1 and HDAC (histone deacetylase) to inhibit IL-17A gene expression (119). This competition for the NFAT binding site also occurs at the promoter of IL-2, a known primary 1,25(OH)_2_D_3_ target gene, suggesting that this may be a general mechanism that also applies to other NFAT-regulated genes (181). Recruitment of HDAC indicates that epigenetic regulation is also important in the inhibition of IL-17A by 1,25(OH)_2_D_3_, especially given the relative epigenetic instability of the IL-17A gene locus (194). Aside from this direct regulation of IL-17A, other mechanisms have also been proposed. One study showed that CHOP is crucial for the inhibitory effect of 1,25(OH)_2_D_3_, while a second study indicated IRF8 to be important (112, 122). Yet another study indicated that VDR forms a complex with VDR, RXR, HDAC2, and Smad3 to inhibit Smad7 transcription, thereby preventing IL-17A production (124). Of note, TGFβ is the cytokine that induces Smad3 and Erk, leading to this inhibition of IL-17A, but it is also the cytokine responsible for inducing the VDR (121). How these mechanisms relate to each other remains to be investigated.

##### Th17.1 Cells

Before the discovery of Th17 cells, it was thought that Th1 cells, characterized by expression of IFNγ, T-bet, and CXCR3, were the major drivers of the autoimmune response. The finding that IL-23, and not IL-12, was required for experimental autoimmunity, at first completely shifted the viewpoint toward Th17 cells as the pathogenic drivers of autoimmunity. However, lately more and more studies indicate that the subdivision into Th17 and Th1 is not as linear as previously assumed. Upon stimulation by IL-12 or TNFα, Th17 cells can become double producers of IL-17A and IFNγ or even shift toward high IFNγ production with little or no IL-17A. Since these latter cells still express CCR6 and RORC, together with T-bet and CXCR3, they are called non-classic Th1 or Th17.1 cells (195). Currently, it is hypothesized that the Th17.1 cells are more pathogenic than Th17 cells in autoimmune diseases, because they are enriched at the sites of inflammation in several diseases (196, 197).

Interestingly, we have shown that in CCR6^+^ cells, which includes Th17 and Th17.1 cells, 1,25(OH)_2_D_3_ reduces the frequency of IFNγ^+^, IL-17A^+^, and IFNγ^+^ IL-17A^+^ cells (120). This suggests that 1,25(OH)_2_D_3_ can inhibit Th cell pathogenicity in autoimmunity via the inhibition of Th17 and Th17.1 cells. A similar effect was found in the CD4^+^ T cells of SLE patients supplemented with 10,400 IU cholecalciferol for 6 months (198). Other supplementation studies have not addressed the combined or single expression of IFNγ and IL-17A, but the results on total IL-17A^+^ or total IFNγ^+^ cells are ambiguous (109, 116, 117).

##### Regulatory T Cells

In contrast to the pro-inflammatory Th subsets mentioned above, regulatory T cells, or Tregs, suppress the immune response. Tregs express FoxP3, the anti-inflammatory cytokines IL-10 and TGFβ, the inhibitory co-receptor CTLA4, and a high level of CD25. They exert immunomodulatory effects on other immune cells such as macrophages, DCs, CD8^+^ T cells, and also other CD4^+^ T cells, thereby maintaining immune homeostasis. Their essential role in preventing autoimmunity is demonstrated in patients with a mutation in FoxP3. These patients are suffering from the IPEX syndrome, which is characterized by massive autoimmunity (199). In the autoimmune diseases discussed here, it is hypothesized that an imbalance between pro-inflammatory T cells, such as Th17 or Th17.1, and Tregs underlies the immune pathogenesis. 1,25(OH)_2_D_3_ may act by restoring this balance and thereby restoring immune homeostasis.

Indeed, 1,25(OH)_2_D_3_ induces FoxP3^+^ Tregs in the spleen, lymph nodes, and spinal cord of EAE mice (119, 124). Additionally, without IL-10 or IL-10-mediated signaling, 1,25(OH)_2_D_3_ cannot inhibit EAE (200). In *in vitro* cultures of Tregs, either obtained via *in vitro* polarization or sorted from peripheral blood, 1,25(OH)_2_D_3_ induces the production of IL-10, but not FoxP3 (114, 201, 202). Polarized Tregs express a higher level of Treg-associated markers such as CTLA4, PD1, and CD25 and their suppressive capacity is enhanced by 1,25(OH)_2_D_3_ (202). Also, the suppressive capacity of Tregs is positively correlated with the serum 25(OH)D_3_ level in MS patients (203). However, when sorted Tregs are used, 1,25(OH)_2_D_3_ does not further enhance their suppressive capacity (114, 201). This suggests that 1,25(OH)_2_D_3_ optimizes Treg function in order to suppress autoimmunity.

Interestingly, 1,25(OH)_2_D_3_ also induces IL-10 production when CD4^+^ cells are cultured under neutral conditions, and even further in the presence of Th17 polarizing cytokines. Furthermore, in these cultures, 1,25(OH)_2_D_3_ also induces FoxP3 and CTLA4, while enhancing the suppressive capacity of the cells (113, 118, 119, 121, 122, 124, 125). Because 1,25(OH)_2_D_3_ inhibits Th17 polarization while inducing IL-10 in these cultures, it was postulated that 1,25(OH)_2_D_3_ may inhibit Th17 activity via IL-10 induction. However, IL-10 is dispensable for the inhibition of IL-17A, suggesting that Th17 inhibition and Treg induction are two independent mechanisms of 1,25(OH)_2_D_3_ (110).

On a molecular level, three mechanisms have been proposed by which 1,25(OH)_2_D_3_ can stimulate a Treg-like phenotype even under Th17 polarizing conditions. First, the VDR can bind to three VDREs in the conserved non-coding sequence of the FoxP3 promoter, thereby directly controlling FoxP3 transcription (119, 125). The second mechanism is by reversing the inhibitory effect of Th17 polarizing cytokines on CTLA4, leading to upregulation of CTLA4 (121). Finally, 1,25(OH)_2_D_3_ induces the expression of IDO, which increases the number of Tregs (76). The latter finding is interesting, since IDO was also reported to be important for the induction of tDCs (see Dendritic Cells) (102), suggesting it might be a general target of 1,25(OH)_2_D_3_ in the immune system.

Although the *in vitro* data demonstrate that 1,25(OH)_2_D_3_ induces Treg cells, not all cholecalciferol supplementation studies find an effect on Tregs. Several studies suggest an increase in the proportion or number of Treg cells based on surface marker expression (109, 116, 204) or based on IL-10 production (50, 117). However, another study did not find this induction in Treg cells (63), and Treg suppressive function is unaffected by cholecalciferol supplementation (117).

Overall, in CD4^+^ T cells, 1,25(OH)_2_D_3_ inhibits the pro-inflammatory Th cell functions while stimulating Treg activity. These effects are observed under both healthy and pathogenic conditions, such as in patients with autoimmune diseases (201). Therefore, restoring the disturbed balance between effector T cells and Treg cells may underlie the beneficial effects of 1,25(OH)_2_D_3_ on autoimmunity.

#### CD8^+^ Cytotoxic T Cells

In addition to CD4^+^ T cells, cytotoxic CD8^+^ T cells comprise the second important class within the T cells. These cells contribute to the immune response by inducing apoptosis in abnormal cells, for example, in case of infection or uncontrolled growth in cancer. In addition, they modulate other immune cells by secreting cytokines (205). Although the role of CD8^+^ T cells in autoimmune diseases is not as well characterized as the role of CD4^+^ T cells, various studies indicate that they play a role in disease pathogenesis. For example, myelin-specific CD8^+^ T cells induce EAE in mice, with characteristics of human MS that are not conferred by myelin-specific CD4^+^ T cells (206, 207). Similarly, hsp60-specific CD8^+^ T cells induce autoimmune intestinal inflammation (208). More recently, it was shown that IL-17A^+^ CD8^+^ T cells are enriched in the synovial fluid of psoriatic arthritis patients. These cells do not express cytolytic markers, but their levels are positively correlated with markers of disease activity (209). Since CD8^+^ T cells have a higher expression of VDR than CD4^+^ T cells (171), CD8^+^ T cells may also be a target for 1,25(OH)_2_D_3_ in the suppression of autoimmunity.

Indeed, adoptive transfer of VDR^−/−^ CD8^+^ T cells in Rag-deficient mice induces intestinal inflammation. When VDR^−/−^ IL-10^−/−^ CD8^+^ T cells are transferred, the intestinal inflammation is even worse and leads to wasting disease (79). The increased proliferation of VDR^−/−^ CD8^+^ T cells, even in the naive state, suggests that VDR-induced signaling is required for maintaining quiescence of these cells. Thereby 1,25(OH)_2_D_3_ prevented hyperactivation of CD8^+^ T cells and subsequent autoimmune pathology in diseases such as CD (79). In addition to maintaining quiescence, 1,25(OH)_2_D_3_ also inhibits the secretion of IFNγ and TNFα by activated CD8^+^ T cells (80). Finally, topical treatment with calcipotriol decreases the frequency of IL-17A^+^ CD8^+^ cells in psoriatic lesions, which is interesting in light of the correlations between these cells and disease activity in psoriatic arthritis (82, 209).

Aside from modulating the activity of the classical CD8^+^ T cells to reduce autoimmunity, 1,25(OH)_2_D_3_ is also important in the development of CD8αα^+^ T cells. CD8αα^+^ T cells are self-reactive cells that have a regulatory function by maintaining homeostasis in the gut. In VDR^−/−^ mice, the number of these cells is reduced, which may explain the susceptibility of these animals to intestinal inflammation (81).

It is important to note that the effect of 1,25(OH)_2_D_3_ is not mediated via the CD8^+^ T cells in every autoimmune disease, since they were dispensable for the attenuation of EAE by 1,25(OH)_2_D_3_ (174). However, it seems that in IBD and psoriatic arthritis, the CD8^+^ T cells are target for 1,25(OH)_2_D_3_. It will be of great interest to determine what the role of the CD8^+^ T cells is in the effect of 1,25(OH)_2_D_3_ on other autoimmune diseases. This will not only provide insight into the mechanisms behind the effect of vitamin D but also about the differences in pathogenesis in the various autoimmune diseases.

#### Unconventional T Cells

Next to the traditional CD4^+^ and CD8^+^ T cells, there are also cells expressing the TCR but lacking both CD4 and CD8. These so-called unconventional T cells have a less diverse TCR repertoire and they are not restricted to MHC class I or II. The unconventional T cells include mucosal-associated invariant T (MAIT) cells, TCRγδ T cells and natural killer T (NKT) cells.

Although MAIT cells have been implicated to be suppressive in autoimmunity, as reviewed by Godfrey et al. (210), there is currently no data available on the effect of 1,25(OH)_2_D_3_ on these cells.

TCRγδ T cells are rapid responders in the event of an infection with intracellular pathogens, due to their recognition of phosphoantigens. Interestingly, they are pathogenic in autoimmune models like EAE and CIA and they produce a wide range of pro-inflammatory cytokines like IL-17A, IL-17F, GM-CSF, TNFα, and IFNγ (211). There is only one study that investigated the effect of 1,25(OH)_2_D_3_ on the pro-inflammatory activity of these cells. They demonstrated that TCRγδ T cells express the VDR upon activation. In response to 1,25(OH)_2_D_3_, the production of IFNγ and the proliferation of these cells was inhibited (87). Currently, it is thought that the main pathogenic action of the TCRγδ T cells in autoimmunity is the secretion of IL-17A (211). Unfortunately, there are no data available yet that describe the effect of 1,25(OH)_2_D_3_ on this cytokine, or any of the other cytokines secreted by the TCRγδ T cells.

The last subset of unconventional T cells that will be discussed here are the NKT cells. They recognize glycolipid antigens and are thereby involved in the protection against a wide range of pathogens. Upon TCR stimulation, NKT cells can rapidly secrete various pro-inflammatory cytokines, including IL-4, IFNγ, and IL-17A. NKT cells can be divided into type I and type II NKT cells. Type I NKT cells are also called invariant NKT (iNKT) cells due to their invariant TCR. Type II NKT cells have a variable TCR and are therefore called the variant NKT cells. The exact role of NKT cells in the pathogenesis of autoimmune disease is not yet completely clear. They are pathogenic in CIA, but they are protective in EAE, T1D, and SLE (88, 212).

Interestingly, VDR is required in the thymus for the development of functionally mature iNKT cells. Furthermore, the iNKT cells in VDR^−/−^ mice are hyporesponsive to TCR stimulation (89). In addition, the protective effect of 1,25(OH)_2_D_3_ in EAE is partially dependent on iNKT cells, possibly via inducing IL-4 in these cells (88). These data suggest that 1,25(OH)_2_D_3_ promotes a suppressive function of iNKT cells. However, given the two-sided effect of iNKT cells in the different autoimmune diseases, further research is needed to fully examine the effect of 1,25(OH)_2_D_3_ on iNKT cell activity and what this means for each individual disease.

### Innate Lymphoid Cells

Recently, a new group of cells became the center of attention in the field of immunology; the innate lymphoid cells (ILC). ILCs play an important role in tissue repair, tissue homeostasis, and the immune response against bacteria, viruses, and fungi. ILCs can be grouped into three classes as follows: (i) the group 1 ILCs (ILC1) that secrete IFNγ and depend on T-bet expression, (ii) the group 2 ILCs (ILC2) that secrete type 2 cytokines such as IL-5 and IL-13 and depend on GATA3, and (iii) the group 3 ILCs (ILC3) that secrete IL-17A and/or IL-22 and depend on RORC (213).

The ILC1s include natural killer cells, which have been known for a longer time and play a role in the clearance of viruses. Since viral triggers are thought to play a role in the initiation of some autoimmune diseases, the NK cells have been investigated for their role in this context. However, under some circumstances, NK cells are protective, while in others they can be pathogenic as recently reviewed by Poggi and Zocchi (214). Also the data on the effect of 1,25(OH)_2_D_3_ on NK cells are somewhat contradictory. In an NK cell line, 1,25(OH)_2_D_3_ induces the cytolytic killing capacity of NK cells (83), but this effect has not been found in healthy control peripheral blood (84, 85). However, when 1,25(OH)_2_D_3_ is added during the *in vitro* differentiation of NK cells from hematopoietic stem cells, the development of NK cells is impaired and their cytotoxicity and IFNγ production are reduced (84). Interestingly, 1,25(OH)_2_D_3_ specifically inhibits activation, cytotoxic capacity and pro-inflammatory cytokine production in overactivated NK cells in women with recurrent pregnancy losses (85). This supports a hypothesis in which 1,25(OH)_2_D_3_ is not a general inhibitor of the immune response, but rather a regulator of immune homeostasis. Therefore, it is of interest whether this abnormal NK activation is also seen in autoimmune diseases and can be modulated by 1,25(OH)_2_D_3_.

Based on their cytokine signature, it can be hypothesized that in the context of autoimmunity ILC3 cells play a role in disease pathogenesis. Indeed, an increase in ILC3 cells has been demonstrated in the lesional skin of psoriasis patients (215, 216), in the inflamed intestine of CD patients (217), in the peripheral blood of MS patients (218), and in the gut, peripheral blood, bone marrow, and synovial fluid of patients with ankylosing spondylitis (219). Furthermore, ILC3 were shown to be responsible for experimental innate-induced colitis (220). Interestingly, in VDR-KO mice, which are susceptible for colitis, the levels of ILC1 and ILC3 are increased (86). On the other hand, calcipotriol treatment did not affect the frequencies of ILC subsets in psoriatic skin lesions after 2 weeks (82).

Since the research into ILC has only started to expand in recent years, the effects of 1,25(OH)_2_D_3_ on these cells have not been investigated extensively. Current data suggest that 1,25(OH)_2_D_3_ may also have anti-inflammatory effects on these cells, but more studies are required to distinguish the effects on the different subsets and its role in the protective effect of vitamin D in autoimmunity.

### Indirect Immunomodulatory Effects

In the previous sections, we discussed the direct modulatory effects of 1,25(OH)_2_D_3_ on various cells of the immune system. However, 1,25(OH)_2_D_3_ and the VDR also affect tissue-resident cells, such as hepatic and pancreatic stellate cells, and the inflammatory mediators that they secrete (221, 222). This indirect mechanism of immune modulation by 1,25(OH)_2_D_3_ is also relevant in autoimmune diseases. For example, in RA, the interaction between T cells and synovial fibroblasts contributes to disease pathogenesis (190). Therefore, it is also of interest to study the effect of 1,25(OH)_2_D_3_ on the tissue-resident cells in the context of autoimmunity.

Similar to the tissue-resident tissue cells in liver and pancreas, 1,25(OH)_2_D_3_ also directly affects RA synovial fibroblasts. Not only is the IL-1β-induced production of tissue-degrading matrix metalloprotease 1 inhibited, also the infiltration capacity of RA fibroblasts is reduced upon treatment with 1,25(OH)_2_D_3_ (223). But this effect on tissue-resident cells is not only found in the synovial cells. It was also shown that the VDR is required for intestinal homeostasis by limiting the production of IL-6 by epithelial cells through inhibition of the NFκB pathway (224). Finally, 1,25(OH)_2_D_3_ also affects brain pericytes, which may be relevant for MS. The pericytes line the epithelial cells of blood vessels, and in the brain, they are important for maintaining the blood–brain barrier and neuron functioning. Brain pericytes cells produce less pro-inflammatory genes when exposed to 1,25(OH)_2_D_3_ while upregulating anti-inflammatory genes. Interestingly, brain pericytes express Cyp27B1 upon stimulation with TNFα and IFNγ. This indicates that an inflammatory environment promotes the conversion of 25(OH)D_3_ into 1,25(OH)_2_D_3_, which then can dampen the inflammation by modulating the pericytes (225).

Overall, the indirect effects of vitamin D and the VDR on immune cells via tissue-resident cells have been underexposed in the past years. However, if we truly want to understand the molecular mechanisms by which 1,25(OH)_2_D_3_ acts in autoimmune diseases, these effects are very important for future studies.

## Future Directions

In this review, we have discussed the advancements that have been made regarding the clinical effects of vitamin D and the molecular mechanisms that underlie these effects. However, there is still a lot that is unclear at the moment, which will be subject of investigation in the coming years.

### Vitamin D Supplementation

Based on the current data on the effect of vitamin D supplementation, it is still not possible to draw conclusions about the added value for the treatment of autoimmunity. This is due to the low number of trials, small patient numbers and heterogeneity in trial setup. In order to determine the therapeutic value of vitamin D supplementation, there are two big open questions that need to be addressed.

First, it is important to assess what serum 25(OH)D_3_ level is required for a beneficial effect of vitamin D in autoimmune diseases. Based on the requirements for calcium homeostasis, current guidelines indicate that a level below 50 nmol/L corresponds with deficiency, between 50 and 74 nmol/L as insufficiency and above 75 nmol/L as a sufficient 25(OH)D_3_ level (226, 227). However, in the context of autoimmunity, it is not known whether it is enough to correct deficiency or whether we should strive for an even higher serum 25(OH)D_3_ level. Using 75 nmol/L as a cut-off point, Raftery et al. showed that CD patients with sufficient serum 25(OH)D_3_ have significantly higher quality of life and less severe disease as measured by intestinal permeability, LL-37 expression, and CDAI (59). Furthermore, in healthy individuals, the serum 25(OH)D_3_ level is correlated with number of VDR binding sites in CD4^+^ T cells. When they have a level above 75 nmol/L, the VDR binding is enriched near genes associated with autoimmune diseases and Tregs (8). However, clinical trials, either with or without placebo controls, do not consistently find immune modulation regardless of the baseline and endpoint serum 25(OH)D_3_ level (Table 2). It should be noted that these measurements have been done in the peripheral blood or in cells from the peripheral blood, which is not the site of inflammation and therefore may not be the most relevant place to look for immunological effects.

**Table 2:** **Overview of clinical trials looking at immunological parameters after vitamin D supplementation**.

Trial	Disease	Supplementation strategy	Mean baseline 25(OH)D_3_	Mean endpoint 25(OH)D_3_	PBMC	T cells		B cells	Innate immune cells (dendritic cell, NK)	Cytokines and antibodies in serum or plasma
						CD4^+^	CD8^+^
Bock et al. (204)	Healthy	3 months 140,000 IU cholecalciferol monthly or placebo	64 ± 29 nmol/L	~138 nmol/L		Increased% of Tregs				

Smolders et al. (117), Knippenberg et al. (170), Peelen et al. (182)	Multiple sclerosis (MS)	12 weeks 20,000 IU cholecalciferol daily (no placebo group)	50 (31–175) nmol/L	308 (151–535) nmol/L		No difference in % or function of Tregs, either naive or memory. Increased production of IL-10 and decreased IL-17A/IL-4 ratio in T cells from PBMC cultures	No relation between % IL-10^+^ or IL-17^+^ CD8^+^ and serum 25(OH)D_3_ No change in% IL-10^+^ or IL-17^+^ CD8^+^	No difference in %, # or differentiation status of circulating B cells		No difference in BAFF No change in immunoglobulins

Kimball et al. (228)	MS	Dose escalation: up to 280,000 IU/week in 23 weeks, stay 6 weeks, then reduce to 0 in 20 weeks, then 3 weeks without [trial: Burton et al. (49)]	78 ± 27 nmol/L	179 ± 76 nmol/L	Decreased PBMC proliferation in response to certain MS-associated antigens					

Mosayebi et al. (50)	MS	6 months 300,000 IU cholecalciferol or placebo i.m. monthly	~25 nmol/L	~140 nmol/L	Decreased PBMC proliferation upon PHA stimulation. No difference in IFNγ, but increase in IL-10 and TGFβ production in these cultures					

Sotirchos et al. (198)	MS	6 months 10,400 or 800 IU cholecalciferol daily	10,400: 68 ± 22 nmol/L 800: 70 ± 21 nmol/L	10,400: +87 (63–112) nmol/L compared to baseline 800: +17 (3–34) nmol/L compared to baseline		High dose, but not low dose, decreases % IL-17^+^, but not % IFNγ^+^ or % IFNγ^+^ IL-17^+^ High dose, but not low dose, decreases % of EM and CD161^+^, while decreasing % of CM and naïve % IL-17^+^ is correlated with % EM For every 12.5 nmol/L increase in serum 25(OH)D_3_, the % IL-17^+^ CD4^+^ decreases by 1% (when serum 25(OH)D_3_ increases more than 45 nmol/L)	High dose, but not low dose, decreases CD85j^+^			

Bendix-Struve et al. (229), Bartels et al. (143)	Crohn’s disease (CD)	1 year placebo vs. 1,200 IU cholecalciferol daily [trial Jørgensen et al. (57)]	33 (16–66) nmol/L	118 (62–154) nmol/L		Over time decrease of IL-6 production is prevented upon supplementation Increased CD4^+^ proliferation is inversely correlated with the IL-10 production			MoDCs have decreased IL-10, IL-6, IL-8, and IL-1β, CD80, and HLA-DR. The allogeneic stimulatory capacities of moDCs are unaffected	

Yang et al. (230)	CD	24 weeks, start with 1,000 IU cholecalciferol daily, increase to 5,000 IU daily or until serum 25(OH)D_3_ is 100 nmol/L (no placebo group)	40 ± 25 nmol/L	113 ± 48 nmol/L						No change in IL-17, TNFα, or IL-10

Gabbay et al. (63)	T1D	18 months 2,000 IU cholecalciferol daily or placebo	66 ± 16 nmol/L	152 ± 54 nmol/L		No change in % Tregs				No difference in IL-12, TNFα, CXCL10, or IL-10, but close-to-significant increase of CCL2 after 12 months (not after 18 months)

Terrier et al. (109)	Systemic lupus erythematosus (SLE)	4 weeks 100,000 IU cholecalciferol weekly, then 6 months 100,000 IU monthly (no placebo group)	47 ± 17 nmol/L	129 ± 35 nmol/L		No change in total % or # Increase in # naive at 6 months, but not %. No change in other activation stages Increase in % and # of Tregs, aTregs, and rTregs. Increase of % CTLA4^+^ and GITR^+^, but not LAP^+^ Tregs Decrease in % of Th1 and Th17 at 2 months, but only of Th1 at 6 months. No change in Th2	No change in total% or #. Decrease in % effector memory at 2 and 6 months, but not #. No change in other activation stages Decrease in IFNγ^+^ at 2 months	Decrease in % and # after 2 months, but after 6 months only in % Increase in MZ% and # after 6 months. Decrease in % and # DN after 6 months. No change in naive or CS B cells	No change in % or # of NK cells	Anti-dsDNA decreased

Abou-Raya et al. (65)	SLE	12 months placebo vs. 2,000 IU cholecalciferol daily	50 ± 41 nmol/L	95 ± 41 nmol/L						Decrease in IL-1β, IL-6, IL-18, and TNFα Decrease in anti-dsDNA, anti-Sm, and C4, but not anticardiolipin IgG or IgM

Piantoni et al. (116), Andreoli et al. (231)	SLE	12 months 25,000 IU cholecalciferol monthly (standard regime, SR) or 300,000 IU at baseline followed by 50,000 IU monthly (intensive regime, IR), compared with healthy control immune parameters	SR: 79 (20–211) nmol/L IR: 80 (47–188) nmol/L	SR: 68 nmol/L IR: 96 nmol/L		Upon SR increase in % and [ ] of iTreg but not tTreg. In IR increased % iTreg and % tTreg, but not [ ]. In SR and IR increase in [ ] highly experienced Tmem, but only in % in SR Increase in total CD4% in SR and IR, but only in [ ] in IR No change in % of IL-17^+^, IFNγ^+^, or IL-4^+^ CD4^+^ T cells after SR and IR	Increase in % but not [ ] of CD8^+^ in SR and IR. No change in % of IL-17^+^, IFNγ^+^, or IL-4^+^ CD8^+^ cells after both SR and IR, but in IR a decreased IFNγ/IL-4 ratio			No difference in anti-dsDNA between SR and IR

*aTreg, activated memory regulatory T cells; BAFF, B-cell activating factor; CM, central memory; CS, class-switched memory; DN, double negative; EM, effector memory; iTreg, induced regulatory T cells; IU, international units; moDC, monocyte-derived dendritic cell; MZ, marginal zone; rTreg, resting regulatory T cells; TE, terminal effector; tTreg, thymic regulatory T cells; #, number; [ ], concentration*.

The second question that is still matter of debate is in what form and dosage vitamin D should be supplemented. In the experimental autoimmune models, animals are mostly supplemented with a high dose of 1,25(OH)_2_D_3_, but in humans, this strategy may lead to hypercalcemia. Therefore, most clinical trials use cholecalciferol as the form of choice, although some use 1,25(OH)_2_D_3_ or less calcemic analogs like alfacalcidol. Of note, a study comparing the effects of alfacalcidol [analog for 1,25(OH)_2_D_3_] with colecalciferol (analog for cholecalciferol) indicates that in the short term alfacalcidol might be more effective, but this effect disappears after 12 months (232). Analogs like calcipotriol that are used in the topical treatment of psoriasis have not been tested in the other autoimmune diseases that were discussed here. Other analogs have been developed, which show equal or better immunomodulatory potential and have been successfully used in experimental autoimmune diseases (201, 233–237). The only analog that was used in clinical trials was alfacalcidol, mainly in type 1 diabetes patients (Table 1). However, the effects of alfacalcidol do not seem better than calcitriol, and at the same dosage, there were no severe side effects from either alfacalcidol or calcitriol (61, 62, 64). More research into the actual effects of vitamin D analogs on human autoimmune disease is required for establishing whether these analogs can be used safely and effectively. Furthermore, in the clinical trials performed so far, there were no serious adverse events after cholecalciferol supplementation. Therefore, it is important to establish the added value of the vitamin D analogs compared to cholecalciferol supplementation. Currently, cholecalciferol is the most used supplementation form in clinical practice. Vitamin D supplementation guidelines indicate a maximum safe dose of 4,000 IU cholecalciferol/day for healthy adults (226). However, no adverse effects were found with dosages of up to 50,000 IU cholecalciferol weekly for 12 weeks, or 100,000 IU weekly for 1 month followed by 100,000 IU monthly for 5 months (54, 109, 117). Interestingly, the dose-escalation regime used by Burton et al. and 20,000 IU weekly by Smolders et al. did not elicit hypercalcemia despite reaching a serum 25(OH)D_3_ level of 400 and 380 nmol/L, respectively (49, 117).

In considering the best strategy for cholecalciferol supplementation, it should also not be forgotten that 1,25(OH)_2_D_3_ may have a synergistic effect with other treatments. For example, *in vitro* studies have shown that 1,25(OH)_2_D_3_ synergizes with retinoic acid (an active vitamin A metabolite) or dexamethason in the inhibition of Th17 pathogenicity (115, 238). Also in monocytes, the combination of dexamethasone and 1,25(OH)_2_D_3_ has added effects over the compounds separately, partially because 1,25(OH)_2_D_3_ enhances the effects of the glucocorticoid receptor (239, 240). Furthermore, we have previously shown that 1,25(OH)_2_D_3_ has an added effect on TNFα blockade in inhibiting the pro-inflammatory loop between Th17 cells and RASF in RA, suggesting that vitamin D combined with anti-TNFα could yield a better treatment response in the treatment of RA patients (120). Finally, combining 1,25(OH)_2_D_3_ with lovastatin has an added therapeutic effect on EAE. This is due to the inhibition of RhoA-ROCK signaling in autoreactive T cells, leading to decreased expression of Cyp24A1 and thereby less inactivation of 1,25(OH)_2_D_3_ (241). Altogether, these data indicate that it may be worthwhile to investigate the addition of cholecalciferol to current treatments like anti-TNFα, or to combine cholecalciferol with, for example, retinoic acid or statins. Due to the synergy between 1,25(OH)_2_D_3_ and these already approved drugs, a lower dose of cholecalciferol may be sufficient for achieving beneficial clinical effects.

Currently, several clinical trials are ongoing and recruiting patients in MS (http://clinicaltrials.gov identifier NCT01490502), RA (NCT02243800), and IBD (NCT02704624, NCT01046773, NCT02208310) for which the results are expected in the coming 3–5 years. Hopefully, they can provide more insight into the answers on these remaining questions. However, to firmly establish the added value of cholecalciferol supplementation, large multicenter trials are required. Ideally, in these trials, the patients should be randomized into different treat-to-target arms, in which every arm has a target 25(OH)D_3_ serum level, such as 75, 100, and 150 nmol/L. Since the effect of cholecalciferol alone is probably not sufficient to control disease activity, patients should receive standard care following pre-defined, harmonized treatment protocols in addition to the cholecalciferol supplementation.

### Molecular Mechanisms Underlying Immunomodulation

In addition to the studies where cholecalciferol has been supplemented, attention has also focused on understanding the immunomodulatory effects of 1,25(OH)_2_D_3_ on a cellular level. Based on the current knowledge, 1,25(OH)_2_D_3_ reduced the pathogenicity of DCs, macrophages, CD4^+^ T cells, CD8^+^ T cells, and B cells. Similar effects have been observed in γδ T cells, iNKT cells, and ILCs, but more research is necessary to confirm these data (see section 5). It should be noted that 1,25(OH)_2_D_3_ does not merely work as an anti-inflammatory agent. Instead, 1,25(OH)_2_D_3_ assists in maintaining the balance between a pro- and anti-inflammatory state and is thereby able to restore the disturbed balance that is associated with autoimmunity.

This balancing effect of 1,25(OH)_2_D_3_ is best illustrated in monocytes and macrophages, where it has pro-inflammatory effects in the early stages of activation but later shifts to an anti-inflammatory state (242). Therefore, it is interesting to study the effects of 1,25(OH)_2_D_3_ in more detail in the various stages of differentiation and activation from monocyte to macrophage. The Carlberg lab has performed ChIP-seq experiments in the monocytic THP-1 cell line at early time points (5). Detailed studies have revealed several primary target genes such as ASAP2 and THBD (243–245), but also identified Bcl6 as a primary target that mediates important secondary responses (246). Next to the primary target genes, combining the ChIP-seq dataset with publically available ChIA-PET and FAIRE-seq datasets has improved the knowledge on VDR binding kinetics (247, 248).

This is just an example of how next-generation sequencing techniques can be combined to yield more understanding of the molecular mechanisms behind the effects of 1,25(OH)_2_D_3_. Since it has already been shown that 1,25(OH)_2_D_3_ has different effects on every cell type, even closely related cell types such as Th1 and Th17 (110), it will be interesting to study VDR DNA binding and identify primary target genes in separate cell types. This will give insight into the similarities and differences between the effects of 1,25(OH)_2_D_3_ on each cell, and what will be important to balance the immune response in patients with autoimmune diseases.

## Conclusion

Although various studies have shown a beneficial effect of cholecalciferol supplementation in autoimmune diseases, there are also studies that do not find any effect on disease parameters. This might be due to the supplementation strategy or the subjects included in the study, which are issues that should be addressed in properly designed multicenter clinical trials.

However, it is also possible that systemic cholecalciferol supplementation is not sufficient to establish effects in every patient. Therefore, another way to use the immunomodulatory effects of vitamin D to the advantage of patients with autoimmune diseases is to mimic the effects by targeting important pathways within immune cells. In order to do this, it is crucial to understand the working mechanisms of 1,25(OH)_2_D_3_. In the coming years, attention should be paid toward unraveling these molecular mechanisms to optimize the therapeutic potential of vitamin D.

## Author Contributions

WD has performed literature research, designed the review layout, and written the review. EC has designed the review layout, contributed to the clinical section, and revised the manuscript. JH has designed the review layout and revised the manuscript. EL has designed the review layout, contributed to the molecular section, and revised the manuscript.

## Conflict of Interest Statement

The authors declare that the research was conducted in the absence of any commercial or financial relationships that could be construed as a potential conflict of interest.
